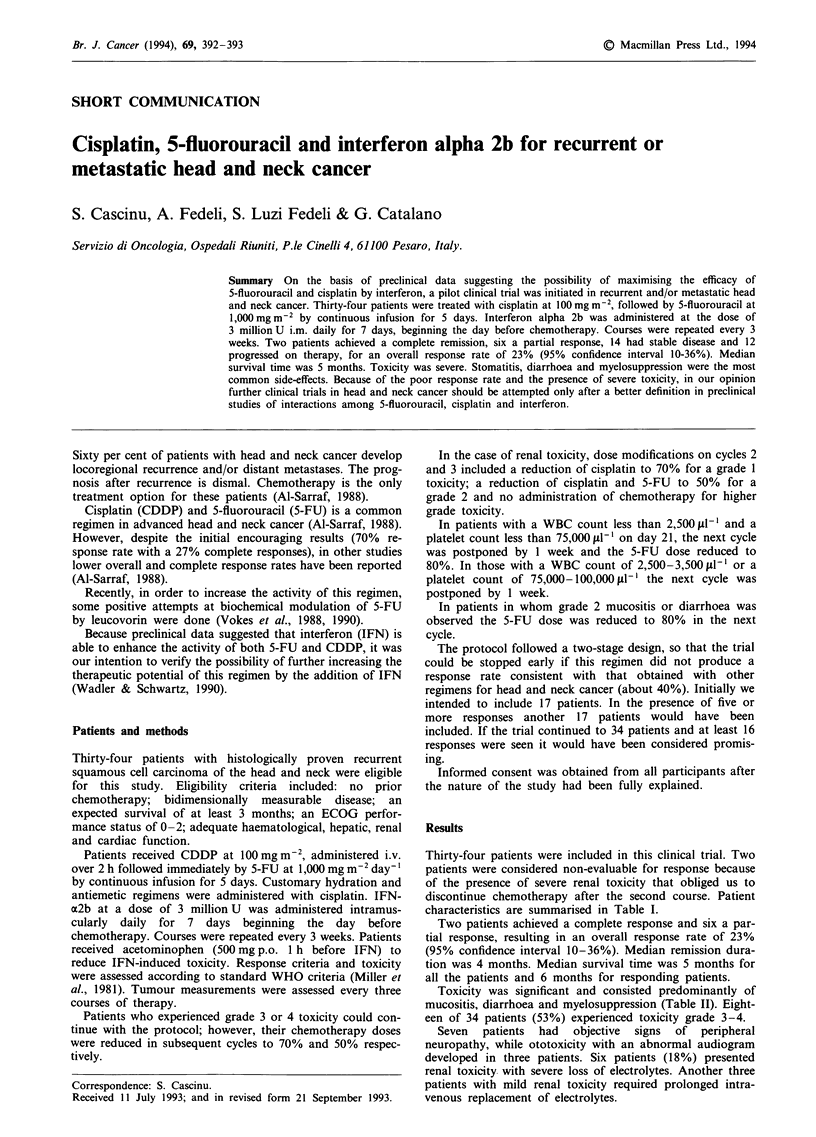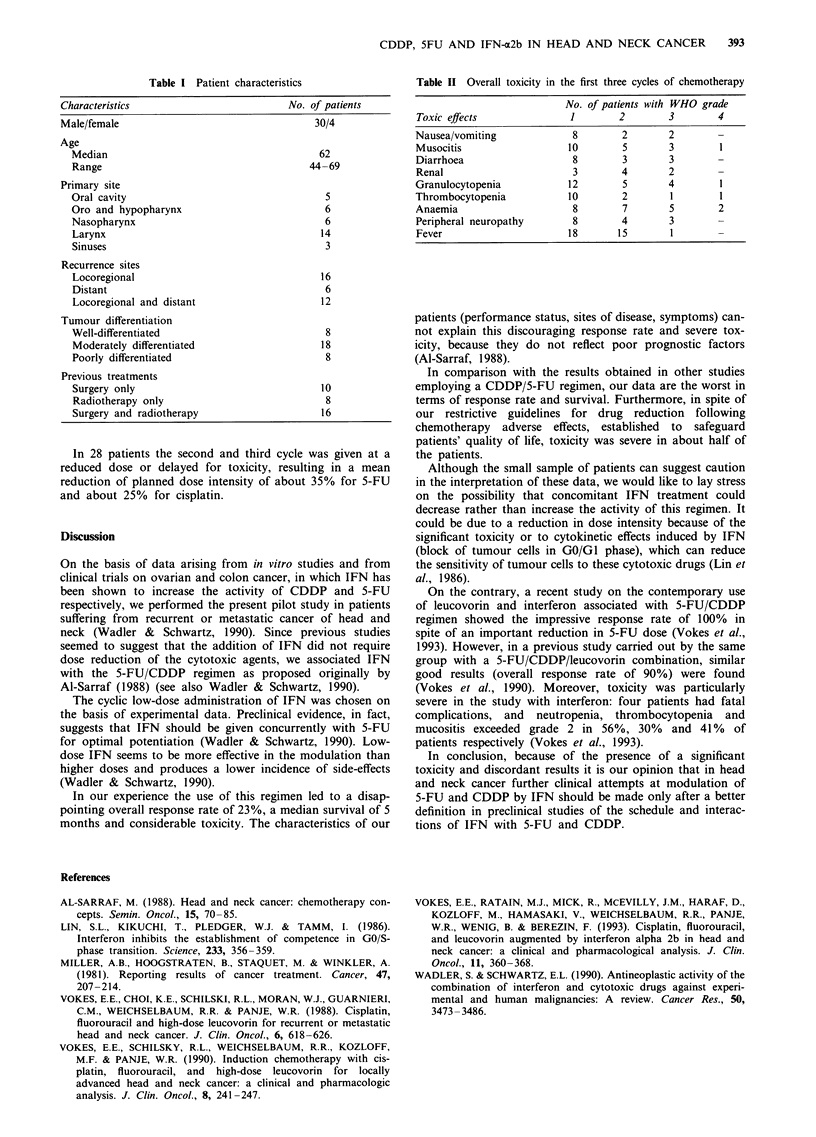# Cisplatin, 5-fluorouracil and interferon alpha 2b for recurrent or metastatic head and neck cancer.

**DOI:** 10.1038/bjc.1994.72

**Published:** 1994-02

**Authors:** S. Cascinu, A. Fedeli, S. Luzi Fedeli, G. Catalano

**Affiliations:** Servizio di Oncologia, Ospedali Riuniti, Pesaro, Italy.

## Abstract

On the basis of preclinical data suggesting the possibility of maximising the efficacy of 5-fluorouracil and cisplatin by interferon, a pilot clinical trial was initiated in recurrent and/or metastatic head and neck cancer. Thirty-four patients were treated with cisplatin at 100 mg m-2, followed by 5-fluorouracil at 1,000 mg m-2 by continuous infusion for 5 days. Interferon alpha 2b was administered at the dose of 3 million U i.m. daily for 7 days, beginning the day before chemotherapy. Courses were repeated every 3 weeks. Two patients achieved a complete remission, six a partial response, 14 had stable disease and 12 progressed on therapy, for an overall response rate of 23% (95% confidence interval 10-36%). Median survival time was 5 months. Toxicity was severe. Stomatitis, diarrhoea and myelosuppression were the most common side-effects. Because of the poor response rate and the presence of severe toxicity, in our opinion further clinical trials in head and neck cancer should be attempted only after a better definition in preclinical studies of interactions among 5-fluorouracil, cisplatin and interferon.


*Br. J. Cancer* (1994), **69**, 392–393 © Macmillan Press Ltd., 1994

SHORT COMMUNICATION

### Cisplatin, 5-fluorouracil and interferon alpha 2b for recurrent or metastatic head and neck cancer

S. Cascinu, A. Fedeli, S. Luzi Fedeli & G. Catalano

*Servizio di Oncologia, Ospedali Riuniti, P.le Cinelli 4, 61100 Pesaro, Italy.*

**Summary** On the basis of preclinical data suggesting the possibility of maximising the efficacy of 5-fluorouracil and cisplatin by interferon, a pilot clinical trial was initiated in recurrent and/or metastatic head and neck cancer. Thirty-four patients were treated with cisplatin at 100 mg m$^{-2}$, followed by 5-fluorouracil at 1,000 mg m$^{-2}$ by continuous infusion for 5 days. Interferon alpha 2b was administered at the dose of 3 million U i.m. daily for 7 days, beginning the day before chemotherapy. Courses were repeated every 3 weeks. Two patients achieved a complete remission, six a partial response, 14 had stable disease and 12 progressed on therapy, for an overall response rate of 23% (95% confidence interval 10-36%). Median survival time was 5 months. Toxicity was severe. Stomatitis, diarrhoea and myelosuppression were the most common side-effects. Because of the poor response rate and the presence of severe toxicity, in our opinion further clinical trials in head and neck cancer should be attempted only after a better definition in preclinical studies of interactions among 5-fluorouracil, cisplatin and interferon.

Sixty per cent of patients with head and neck cancer develop locoregional recurrence and/or distant metastases. The prognosis after recurrence is dismal. Chemotherapy is the only treatment option for these patients (Al-Sarraf, 1988).

Cisplatin (CDDP) and 5-fluorouracil (5-FU) is a common regimen in advanced head and neck cancer (Al-Sarraf, 1988). However, despite the initial encouraging results (70% response rate with a 27% complete responses), in other studies lower overall and complete response rates have been reported (Al-Sarraf, 1988).

Recently, in order to increase the activity of this regimen, some positive attempts at biochemical modulation of 5-FU by leucovorin were done (Vokes *et al.*, 1988, 1990).

Because preclinical data suggested that interferon (IFN) is able to enhance the activity of both 5-FU and CDDP, it was our intention to verify the possibility of further increasing the therapeutic potential of this regimen by the addition of IFN (Wadler & Schwartz, 1990).

### Patients and methods

Thirty-four patients with histologically proven recurrent squamous cell carcinoma of the head and neck were eligible for this study. Eligibility criteria included: no prior chemotherapy; bidimensionally measurable disease; an expected survival of at least 3 months; an ECOG performance status of 0–2; adequate haematological, hepatic, renal and cardiac function.

Patients received CDDP at 100 mg m$^{-2}$, administered i.v. over 2 h followed immediately by 5-FU at 1,000 mg m$^{-2}$ day$^{-1}$ by continuous infusion for 5 days. Customary hydration and antiemetic regimens were administered with cisplatin. IFN-α2b at a dose of 3 million U was administered intramuscularly daily for 7 days beginning the day before chemotherapy. Courses were repeated every 3 weeks. Patients received acetominophen (500 mg p.o. 1 h before IFN) to reduce IFN-induced toxicity. Response criteria and toxicity were assessed according to standard WHO criteria (Miller *et al.*, 1981). Tumour measurements were assessed every three courses of therapy.

Patients who experienced grade 3 or 4 toxicity could continue with the protocol; however, their chemotherapy doses were reduced in subsequent cycles to 70% and 50% respectively.

Correspondence: S. Cascinu.
Received 11 July 1993; and in revised form 21 September 1993.

In the case of renal toxicity, dose modifications on cycles 2 and 3 included a reduction of cisplatin to 70% for a grade 1 toxicity; a reduction of cisplatin and 5-FU to 50% for a grade 2 and no administration of chemotherapy for higher grade toxicity.

In patients with a WBC count less than 2,500 μl$^{-1}$ and a platelet count less than 75,000 μl$^{-1}$ on day 21, the next cycle was postponed by 1 week and the 5-FU dose reduced to 80%. In those with a WBC count of 2,500–3,500 μl$^{-1}$ or a platelet count of 75,000–100,000 μl$^{-1}$ the next cycle was postponed by 1 week.

In patients in whom grade 2 mucositis or diarrhoea was observed the 5-FU dose was reduced to 80% in the next cycle.

The protocol followed a two-stage design, so that the trial could be stopped early if this regimen did not produce a response rate consistent with that obtained with other regimens for head and neck cancer (about 40%). Initially we intended to include 17 patients. In the presence of five or more responses another 17 patients would have been included. If the trial continued to 34 patients and at least 16 responses were seen it would have been considered promising.

Informed consent was obtained from all participants after the nature of the study had been fully explained.

### Results

Thirty-four patients were included in this clinical trial. Two patients were considered non-evaluable for response because of the presence of severe renal toxicity that obliged us to discontinue chemotherapy after the second course. Patient characteristics are summarised in Table I.

Two patients achieved a complete response and six a partial response, resulting in an overall response rate of 23% (95% confidence interval 10–36%). Median remission duration was 4 months. Median survival time was 5 months for all the patients and 6 months for responding patients.

Toxicity was significant and consisted predominantly of mucositis, diarrhoea and myelosuppression (Table II). Eighteen of 34 patients (53%) experienced toxicity grade 3–4.

Seven patients had objective signs of peripheral neuropathy, while ototoxicity with an abnormal audiogram developed in three patients. Six patients (18%) presented renal toxicity with severe loss of electrolytes. Another three patients with mild renal toxicity required prolonged intravenous replacement of electrolytes.

**Table I** Patient characteristics

| *Characteristics* | *No. of patients* |
|---|---|
| Male/female | 30/4 |
| Age | |
| Median | 62 |
| Range | 44–69 |
| Primary site | |
| Oral cavity | 5 |
| Oro and hypopharynx | 6 |
| Nasopharynx | 6 |
| Larynx | 14 |
| Sinuses | 3 |
| Recurrence sites | |
| Locoregional | 16 |
| Distant | 6 |
| Locoregional and distant | 12 |
| Tumour differentiation | |
| Well-differentiated | 8 |
| Moderately differentiated | 18 |
| Poorly differentiated | 8 |
| Previous treatments | |
| Surgery only | 10 |
| Radiotherapy only | 8 |
| Surgery and radiotherapy | 16 |

In 28 patients the second and third cycle was given at a reduced dose or delayed for toxicity, resulting in a mean reduction of planned dose intensity of about 35% for 5-FU and about 25% for cisplatin.

### Discussion

On the basis of data arising from *in vitro* studies and from clinical trials on ovarian and colon cancer, in which IFN has been shown to increase the activity of CDDP and 5-FU respectively, we performed the present pilot study in patients suffering from recurrent or metastatic cancer of head and neck (Wadler & Schwartz, 1990). Since previous studies seemed to suggest that the addition of IFN did not require dose reduction of the cytotoxic agents, we associated IFN with the 5-FU/CDDP regimen as proposed originally by Al-Sarraf (1988) (see also Wadler & Schwartz, 1990).

The cyclic low-dose administration of IFN was chosen on the basis of experimental data. Preclinical evidence, in fact, suggests that IFN should be given concurrently with 5-FU for optimal potentiation (Wadler & Schwartz, 1990). Low-dose IFN seems to be more effective in the modulation than higher doses and produces a lower incidence of side-effects (Wadler & Schwartz, 1990).

In our experience the use of this regimen led to a disappointing overall response rate of 23%, a median survival of 5 months and considerable toxicity. The characteristics of our patients (performance status, sites of disease, symptoms) cannot explain this discouraging response rate and severe toxicity, because they do not reflect poor prognostic factors (Al-Sarraf, 1988).

**Table II** Overall toxicity in the first three cycles of chemotherapy

| | *No. of patients with WHO grade* | | | |
|---|---|---|---|---|
| *Toxic effects* | *1* | *2* | *3* | *4* |
| Nausea/vomiting | 8 | 2 | 2 | – |
| Musocitis | 10 | 5 | 3 | 1 |
| Diarrhoea | 8 | 3 | 3 | – |
| Renal | 3 | 4 | 2 | – |
| Granulocytopenia | 12 | 5 | 4 | 1 |
| Thrombocytopenia | 10 | 2 | 1 | 1 |
| Anaemia | 8 | 7 | 5 | 2 |
| Peripheral neuropathy | 8 | 4 | 3 | – |
| Fever | 18 | 15 | 1 | – |

In comparison with the results obtained in other studies employing a CDDP/5-FU regimen, our data are the worst in terms of response rate and survival. Furthermore, in spite of our restrictive guidelines for drug reduction following chemotherapy adverse effects, established to safeguard patients' quality of life, toxicity was severe in about half of the patients.

Although the small sample of patients can suggest caution in the interpretation of these data, we would like to lay stress on the possibility that concomitant IFN treatment could decrease rather than increase the activity of this regimen. It could be due to a reduction in dose intensity because of the significant toxicity or to cytokinetic effects induced by IFN (block of tumour cells in G0/G1 phase), which can reduce the sensitivity of tumour cells to these cytotoxic drugs (Lin *et al.*, 1986).

On the contrary, a recent study on the contemporary use of leucovorin and interferon associated with 5-FU/CDDP regimen showed the impressive response rate of 100% in spite of an important reduction in 5-FU dose (Vokes *et al.*, 1993). However, in a previous study carried out by the same group with a 5-FU/CDDP/leucovorin combination, similar good results (overall response rate of 90%) were found (Vokes *et al.*, 1990). Moreover, toxicity was particularly severe in the study with interferon: four patients had fatal complications, and neutropenia, thrombocytopenia and mucositis exceeded grade 2 in 56%, 30% and 41% of patients respectively (Vokes *et al.*, 1993).

In conclusion, because of the presence of a significant toxicity and discordant results it is our opinion that in head and neck cancer further clinical attempts at modulation of 5-FU and CDDP by IFN should be made only after a better definition in preclinical studies of the schedule and interactions of IFN with 5-FU and CDDP.

### References

AL-SARRAF, M. (1988). Head and neck cancer: chemotherapy concepts. *Semin. Oncol.*, **15**, 70–85.

LIN, S.L., KIKUCHI, T., PLEDGER, W.J. & TAMM, I. (1986). Interferon inhibits the establishment of competence in G0/S-phase transition. *Science*, **233**, 356–359.

MILLER, A.B., HOOGSTRATEN, B., STAQUET, M. & WINKLER, A. (1981). Reporting results of cancer treatment. *Cancer*, **47**, 207–214.

VOKES, E.E., CHOI, K.E., SCHILSKI, R.L., MORAN, W.J., GUARNIERI, C.M., WEICHSELBAUM, R.R. & PANJE, W.R. (1988). Cisplatin, fluorouracil and high-dose leucovorin for recurrent or metastatic head and neck cancer. *J. Clin. Oncol.*, **6**, 618–626.

VOKES, E.E., SCHILSKY, R.L., WEICHSELBAUM, R.R., KOZLOFF, M.F. & PANJE, W.R. (1990). Induction chemotherapy with cisplatin, fluorouracil, and high-dose leucovorin for locally advanced head and neck cancer: a clinical and pharmacologic analysis. *J. Clin. Oncol.*, **8**, 241–247.

VOKES, E.E., RATAIN, M.J., MICK, R., McEVILLY, J.M., HARAF, D., KOZLOFF, M., HAMASAKI, V., WEICHSELBAUM, R.R., PANJE, W.R., WENIG, B. & BEREZIN, F. (1993). Cisplatin, fluorouracil, and leucovorin augmented by interferon alpha 2b in head and neck cancer: a clinical and pharmacological analysis. *J. Clin. Oncol.*, **11**, 360–368.

WADLER, S. & SCHWARTZ, E.L. (1990). Antineoplastic activity of the combination of interferon and cytotoxic drugs against experimental and human malignancies: A review. *Cancer Res.*, **50**, 3473–3486.